# The influence of lidocaine topical anesthesia during transesophageal echocardiography on blood methemoglobin level and risk of methemoglobinemia

**DOI:** 10.1007/s10554-015-0608-z

**Published:** 2015-02-07

**Authors:** Dominika Filipiak-Strzecka, Jarosław D. Kasprzak, Marta Wiszniewska, Jolanta Walusiak-Skorupa, Piotr Lipiec

**Affiliations:** 1 Department of Cardiology, Bieganski Hospital, Medical University of Lodz, Kniaziewicza 1/5, 91-347 Lodz, Poland; 2 Department of Occupational Diseases, Nofer Institute of Occupational Medicine, Lodz, Poland

**Keywords:** Methemoglobinemia, Transesophageal echocardiography, Lidocaine, Local anesthesia

## Abstract

Methemoglobinemia is a relatively rare, but potentially life-threating medical condition, which may be induced by application of topical anaesthetic agents commonly used during endoscopic procedure. The aim of our study was to assess the influence of lidocaine used prior to transesophageal echocardiography (TEE) on the blood level of methemoglobin in vivo. Additionally we attempted to establish the occurrence rate of clinically evident lidocaine-induced methemoglobinemia on the basis of data collected in our institution. We retrospectively analyzed patient records from 3,354 TEEs performed in our echocardiographic laboratory over the course of 13 years in search for clinically evident methemoglobinemia cases. Additionally, 18 consecutive patients referred for TEE were included in the prospective part of our analysis. Blood samples were tested before and 60 min after pre-TEE lidocaine anesthesia application. Information concerning concomitant conditions and pharmacotherapy were also obtained. In 3,354 patients who underwent TEE in our institution no cases of clinically evident methemoglobinemia occurred. In the prospective part of the study, none of 18 patients [16 (89 %) men, mean age 63 ± 13] was diagnosed with either clinical symptoms of methemoglobinemia or exceeded normal blood concentration of methemoglobin. Initial mean methemoglobin level was 0.5 ± 0.1 % with mild, statistically (but not clinically) significant rise to 0.6 ± 0.1 % after 60 min (*p* = 0.02). Among the analyzed factors only the relation between the proton pump inhibitors intake and methemoglobin blood level rise was identified as statistically relevant (*p* = 0.03). In adults, pre-TEE lidocaine anesthesia with recommended dosage results in significant increase in methemoglobin blood level, which however does not exceed normal values and does not result in clinically evident methemoglobinemia.

## Introduction

Methemoglobinemia is a relatively rare, but potentially life-threating medical condition, especially if not recognized and treated immediately [1–4]. It is defined by increased concentration of an oxidized form of hemoglobin in which the heme iron exists in the ferric (Fe+3) state [5]. This form of hemoglobin is not only unable to bind oxygen but also shifts the oxygen-hemoglobin dissociation curve to the left and changes its sigmoid shape into a more hyperbolic one, thus impairing oxygen extraction in the tissues [6]. Therefore, excessive replacement of hemoglobin with methemoglobin leads to functional anemia and tissue hypoxia. First symptoms are cyanosis, low pulse oximetric readings, and chocolate-brown color of arterial blood sampling with normal arterial PO_2_ values. Clinically, patients may suffer from shortness of breath, cough and dizziness. In case of severe methemoglobinemia episode, with methemoglobin level exceeding 55 %, patients may develop lethargy, stupor, and deteriorating consciousness. Higher levels (methemoglobin level >70 %) may result in dysrhythmias, circulatory failure, neurological depression, which in extreme cases could be lethal [7]. The diagnosis is established by measuring methemoglobin levels using CO-oximetry in the arterial or venous blood [8].

In normal conditions methemoglobin rarely exceeds the level of 1.5 % of total hemoglobin content, considered as an upper limit of normal value.

The causes of methemoglobinemia may be congenital (hereditary enzymatic disorders) or acquired. The latter may occur after exposure to toxins or drugs. Agents may be divided into direct oxidizers, capable of inducing methemoglobin formation when added to erythrocytes both in vitro or in vivo, and indirect oxidizers, which do not induce methemoglobin formation when exposed to erythrocytes in vitro, but do so after metabolic modification in vivo [9]. The example of indirect oxidizers are local anesthesia agents.

Local anesthetics are routinely administered in nasopharyngeal and oropharyngeal anesthesia, prior to endoscopic procedures [10]. The vast majority of reported methemoglobinemia cases related with exposure to anesthetic drugs occurred after benzocaine administration. Therefore certain authors suggested that benzocaine should no longer be used as topical anesthetic for mucosa [11, 12]. Lidocaine appears to be a safer alternative, however its ability to induce methemoglobin formation remains unknown. The aim of our study was to assess the influence of standard dose of lidocaine used for anesthesia of pharyngeal mucosa prior to transesophageal echocardiographic examination (TEE) on the level of methemoglobin. Additionally we attempted to retrospectively establish the occurrence rate of clinically evident lidocaine-induced methemoglobinemia using a retrospective analysis of the databases in our institution.

## Methods

### TEE protocol

The protocol applied in our institution for pre-TEE oropharygeal anesthesia is based on direct application of 10 % lidocaine spray (2 doses—9.6 mg of pure lidocaine) while the ultrasonographic gel used during the examination does not contain any anesthetics. Saturation is continuously monitored during the procedure by means of finger pulsoximetry.

The majority of patients undergoing TEE in our echo laboratory are hospitalized due to concomitant conditions and remain in our department for at least 24 h. Should the examination be performed as an outpatient procedure, it is followed by 2 h of clinical observation. If no adverse effects occur, patients are discharged after being informed about the obligation to report to the emergency department of our institution if deteriorate clinically.

### Database analysis

We aimed to identify all cases of clinically evident methemoglobinemia ensuing after TEE performed at our institution in the period between January the 1st of 2000 and 14th October 2013. To achieve this we have analyzed the echocardiography database as well as electronic medical records of our department in search of methemoglobinemia diagnosis. To minimize the risk of overlooking the cases of methemoglobinemia which may have occurred after hospital discharge, Emergency Department database was also included in the analysis.

### Prospective analysis

Eighteen consecutive patients [16 (89 %) men, mean age 63 ± 13] hospitalized in our department, with clinical need for TEE, were enrolled in the study. All subject signed an informed consent form. Before the examination onset a questionnaire based clinical history inclusive of age, sex, weight, height, episodes of fever >38 °C within the last 7 days, chronic renal disease, heart failure, ischemic heart disease, nitrate/oral hypoglycemic/proton pump inhibitors pharmacotherapy (which was considered as a potential risk factor of methemoglobinemia in the previous studies [8, 13, 14]), hypersensitivity to local anesthesia or previous episodes of methemoglobinemia was collected from all participants. The sole exclusion criterion was exposure to local anesthesia agents within previous 7 days.

Indication for TEE study and current hemoglobin level (test performed within 24 h) were extracted from medical records.

Fourteen patients were referred to TEE prior to cardioversion in order to rule out the suspicion of thrombus in left atrium appendage (in one case the thrombus was detected in TEE); in three patients TEE was a part of valvular diseases diagnostics, in one patient atrial septum defect was suspected after transthoracic echocardiography examination. None of the patients manifested the clinical symptoms of infective endocarditis. Mean level of hemoglobin in the study population was 14.6 ± 1.7 mg/dl (range 11.2–17.8 mg/dl), mean O_2_ saturation was 97 ± 1 % (range 94–97 %).

One patient reported increase in body temperature >38 °C within 7 days preceding the examination. Chronic renal disease, heart failure, ischemic heart disease were present in medical history of two, three and four patients, respectively. One patient received a chronic long-acting nitrate therapy, two patients were treated with oral hypoglycemics and nine with proton pump inhibitors.

Prior to the previously described topical anesthesia protocol preceding TEE, a blood sample (1 ml anaerobically in a Vaculette Lithium Heparine blood syringe) was taken from each patient.

16 out of 18 patients received sedation (midazolam intravenously, mean dose 3.4 mg, range 2–5 mg).

Having completed the echocardiographic examination, we drew another blood sample from another puncture site after 60 min from lidocaine application. Both blood samples were immediately placed in the cool and dry environment (temperature 5 °C). Samples were tested for methemoglobin level with a Cobas b 211 (Roche Diagnostic) diagnostic workstation. Measurement is based on the relation between characteristic absorption of hemoglobin derivative and the wave length according to Lambert–Beer law. This instrument reports the methemoglobin level to the nearest tenth of a percent.

### Statistical analysis

Data were stored and statistical analyses performed using Statistica version 10.0 (StatSoft Poland, Cracow, Poland) by means of simple descriptive statistics and Wilcoxon and Man-Whitney statistical test. *p* value <0.05 was considered statistically significant.

## Results

### Datebase analysis

The total of 3,354 TEE were performed during the period between 1st January 2000 and 14th October 2013 [1,911 (57 %) men, mean age 56 ± 16]. In 115 (3.4 %) cases the referral diagnosis was infective endocarditis and in 252 (7.5 %) cases there was a thrombus present in heart cavities. No cases of methemoglobinemia could be identified in discussed group.

### Prospective analysis

None of 18 patients revealed clinical symptoms of methemoglobinemia. The post-lidocaine values of methemoglobin level remained in all patients below the upper normal limit for methemoglobin (1.5 %).

**Fig. 1:**
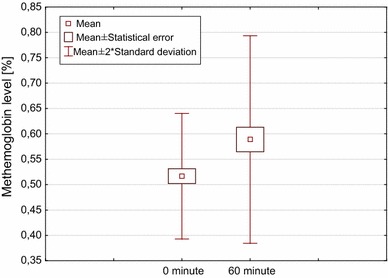
Plot of changes in methemoglobin level before and 60 min after administration of lidocaine

Initial mean methemoglobin level was 0.5 ± 0.1 % (range 0.4–0.6 %) whereas after 60 min it reached the mean level of 0.6 ± 0.1 % (range 0.5–0.9 %) which represented a statistically significant change (*p* = 0.02, Wilcoxon test) (Fig. 1).

Subsequently the correlation between above mentioned clinical variables pharmacotherapy and rise of methemoglobin blood level was tested (Mann–Whitney test, Table 1). Among the analyzed factors only the relationship between the treatment with proton pump inhibitors and methemoglobin blood level rise could be qualified as statistically relevant (mean change in methemoglobin level in patients taking proton pump inhibitors: 0.13 ± 0.12 %, range 0–0.4 %; in the remaining patients: 0.01 ± 0.09 %, range 0.1–0.2 %; *p* value 0.03).

**Table 1:** Characteristics of prospective study population

	Number of patients
Total number of patients	18
Male sex	16 (89 %)
Mean age (years)	63 ± 13
Fever >38 °C within the last 7 days	1 (5.6 %)
Chronic renal disease	2 (11.1 %)
Heart failure	3 (16.7 %)
Ischemic heart disease	4 (22.2 %)
Pharmacotherapy	
Nitrate	1 (5.6 %)
Oral hypoglycemic	2 (11.1 %)
Proton pump inhibitors	9 (50 %)
Hypersensitivity to local anesthesia	0
Previous episodes of methemoglobinemia	0

## Discussion

To the best of our knowledge this study is the first one to prospectively analyze the influence of pre-TEE lidocaine exposure on the blood methemoglobin content and occurrence of clinical methemoglobinemia. On the basis of the results of our prospective analysis combined with retrospective study of our institution databases the following findings may be issued: (1) Pre-TEE exposure to recommended lidocaine doses results in statistically significant increase of methemoglobin blood level which however does not exceed normal values. (2) Lidocaine administered in recommended doses is a relatively safe local anesthetic agent for oropharyngeal topical anesthesia in patients undergoing TEE.

Four types of local anesthetics have been suspected as possible cause of methemoglobinemia: prilocaine, benzocaine, lidocaine, and tetracaine. Its occurrence may be possibly related to a number of clinical factors such as age, dose of medication, enzyme deficiencies, malnutrition, mucosal erosion, hospitalization, sepsis, and anemia [7]. However, during the endoscopic procedures, agents most commonly used for oropharyngeal anesthesia are either benzocaine or lidocaine spray. Systematic reviews published up to date, as well as case-reports, indicate the significantly higher methemoglobinemia occurrence rate related with benzocaine exposure than with lidocaine anesthesia. In a review presenting 242 cases of local anesthesia–related methemoglobinemia 159 (65.7 %) patients were anesthetized with agents containing benzocaine, among which 105 (43.4 %) patients were treated with benzocaine alone. 12 incidents of methemoglobinemia were initially connected with lidocaine application, however only three patients episode could not be attributed with any other cause than topical lidocaine administration [12, 15–17].

In another study analyzing 24,431 patients undergoing endoscopic procedures, no cases of methemoglobinemia occurred among 22,210 patients anesthetized with 4 % lidocaine spray prior to upper gastrointestinal endoscopy/bronchoscopy. Adversely, in the second group consisting of 2,221 patients in whom 20 % benzocaine spray anesthesia was performed prior to TEE, nine cases of clinically significant methemoglobinemia were reported [11], a significant difference in risk.

Similarly, in our population of 3,354 patients undergoing TEE during the last 13 years not a single case of clinically manifesting methemoglobinemia was detected. Due to retrospective character of database analysis there is a theoretical possibility of discarding the events of methemoglobinemia in patients who were not hospitalized after the TEE. However, the number of such patients did not exceed 15 % of total retrospective analysis population. Furthermore, if they had developed clinical methemoglobinemia, their records should have been included into ER source data.

Methemoglobinemia associated with lidocaine has been shown to be caused by metabolites produced from xylidine after it has been hydrolyzed from lidocaine [18]. However, only a part of absorbed lidocaine is hydrolyzed to xylidine. Furthermore, in a recent in vitro study based on the incubation of benzocaine, lidocaine and xylidine with whole human blood and pooled human liver S9, authors have observed that benzocaine produces much more methemoglobin than lidocaine or xylidine [19].

Maximal acceptable dose of lidocaine used subcutaneously or intravenously is 200 mg (4.5 mg/kg of body mass). It could be assumed that during topical mucosal anesthesia the lidocaine dose is not completely absorbed; furthermore, doses routinely used prior to endoscopic procedures are significantly smaller. Patients included in our study received ca. 10 mg of pure lidocaine regardless of body weight (max. 0.15 mg/kg of body mass). Although none of them developed methemoglobinemia, a statistically significant increase in methemoglobin blood level was detected. Thus, it can be assumed that although lidocaine is considered a safe topical mucosa anesthetic, its dose should be carefully adjusted and minimal effective dose should be administered in all cases [20].

Our echocardiography suite has no experience or clinical data regarding the benzocaine anesthesia as it has never been used for the purpose discussed.

In conclusion, pre-TEE lidocaine anesthesia with a dose close to 10 % of maximal accepted produced statistically relevant but clinically insignificant increase in methemoglobin blood level. According to our data lidocaine is a relatively safe topical anesthetic agent. This corresponds well with findings of up-to-date scientific literature suggesting that lidocaine is less likely to cause methemoglobinemia than benzocaine when used during certain endoscopic procedures. Therefore it may be beneficial for the patients to consider lidocaine as a medicine of choice for topical oropharyngeal mucosa anesthesia.

The study protocol was approved by Bioethics Committee of our institution. Therefore the study has been performed in accordance with the ethical standards laid down in the 1964 Declaration of Helsinki and its later amendments. All patients gave their informed consent prior to their inclusion in the study.
